# First Movers in Molecular Detection: Case Comparison on Harnessing Research and Development, Industry, and Entrepreneurship

**DOI:** 10.3389/fmed.2021.639440

**Published:** 2021-03-25

**Authors:** Kenneth B. Yeh, Matt Scullion, Julia M. Michelotti, Gene Olinger

**Affiliations:** ^1^MRIGlobal, Gaithersburg, MD, United States; ^2^BioFire Defense, Salt Lake City, UT, United States

**Keywords:** COVID-19, molecular detection, CRISPR, qPCR, first mover

## Abstract

The current unprecedented COVID-19 pandemic underscores the importance of diagnostic assays in health security preparedness and readiness. Advancing new technologies for rapid molecular detection of high consequence infectious pathogens is an ongoing challenge that requires ingenuity and vision. Sustainment of a robust supply chain for materials and the logistics of timely product delivery further challenge diagnostic kit and device manufacturers. Business economists often characterize technology companies that discover unique breakthroughs in their field and are first to bring related products to market as first movers. From a market perspective, three first mover characteristics include: having the knowledge and capability to address a unique breakthrough, excellent technological leadership, and the ability to capitalize on the opportunity. Current mainstays for molecular detection include using Taq DNA Polymerase enzyme and fluorescent chemistry for quantitative PCR (qPCR). A newer and promising technology uses CRISPR-Cas proteins for nucleic acid detection. Our panel discussion from the 2020 ASM Biothreats conference, which included members from two prototypical first mover companies, explored their respective corporate experiences. Both companies were selected for the discussion based on their revolutionary innovations and similarities in their research and development, corporate culture and trajectory. One company, established over 20 years ago, became a market leader in the biothreat detection market by advancing air thermocycling qPCR across multiple product families. The second company is a rapidly growing start-up and a scientific pioneer in establishing next generation CRISPR technologies. Here we discuss their technology development, product deployment, and customer markets to draw lessons learned for researchers, end users, and funders.

Like the technological revolution and the Industrial Revolution before it, the biological revolution will reshape how we interact with and understand the world around us^1^

- Former United States Senator Cory Gardner.

## Background

**Figure 1:**
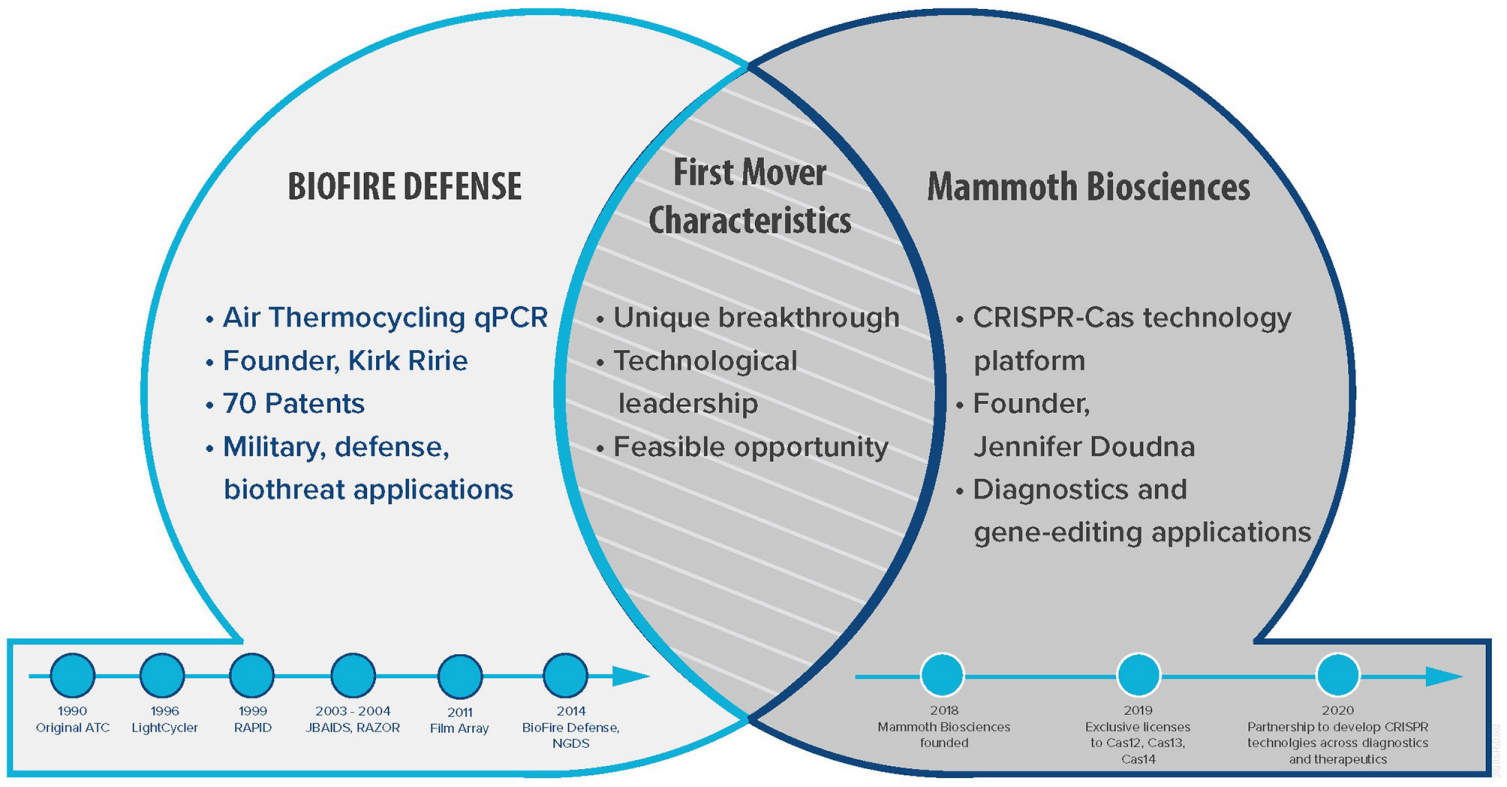
First Movers. This Venn diagram illustrates the two prototypical first mover companies in biotechnology.

As part of a panel discussion at the 2020 American Society for Microbiology's (ASM) Biothreat Conference, we discussed and contrasted experiences on technology and business with representatives from two prototypical first mover companies in the biotechnology industry. The corporate panel participants and major topics are described in Figure 1. This event, inspired by a similar panel discussion on “Molecular Detection in the Field” at the 2019 ASM Biothreats conference, is part of a continued collaboration to develop and publish current findings on the importance of rapidly detecting and identifying high consequence pathogens. Presenting a panel discussion to a diverse audience that includes researchers, end users, funders, and programmers is a unique and important forum to stimulate new ideas (1). Our findings are relevant across science and business sectors especially to reinforce public and private partnerships.

The clinical and biodefense diagnostics markets are each valued at over 1.5 billion dollars per year (2, 3). As a result, a significant number of laboratories are involved in molecular detection including product manufacturers, test and reference laboratories, academic institutes, and commercial research organizations. While maintaining country preparedness and readiness to respond to infectious disease outbreaks—whether intentional (bioterrorism), accidental, or naturally occurring—is typically a national priority, the niche markets created for biodefense, are expensive for commercial entities and may not be profitable due to the relatively low frequency of these events. Real-world events catalyze niche markets as in the case of the 2001 anthrax letter attacks where the United States (US) Government response had the effect of continuing biodefense related activities and establishing that market as an enterprise. Prior to this event, national laboratories and research organizations typically performed research and development (R&D) in biodefense. More recently, the biodefense market attracts those in other fields contributing to an expanded range of products (3). The availability of national biodefense funding for public corporations and private institutes allows them to remain viable in their respective markets (4). Achieving a return on investment is especially challenging for companies that develop cutting-edge technologies and define new market segments involving diagnostic assays, medical countermeasures, and protective equipment. There are additional challenges to take into account when addressing outbreaks caused by emerging and re-emerging pathogens; SARS-CoV, MERS-CoV, Zika virus, and SARS-CoV-2 are prime examples of viruses with significant human and economic impacts.

Detection and identification of microbiological pathogens including bacteria, fungi, and viruses requires the presence of the organism, or some part of it such as nucleic acid material. Pathogenic microbes are often categorized into Risk Groups 1–4 according to their “capability to cause disease in a susceptible human or animal host, virulence as measured by severity of the disease and the availability of effective preventative measures and treatments” (5). Risk Group 3 and 4 pathogens that infect animals, humans, and plants may also be classified as biological threat agents (biothreats) due to the potential for nefarious use (6, 7). Designing molecular detection methods such as qPCR and reverse transcription qPCR (RT-qPCR) relies on an *a priori* knowledge of the biological agent of interest such as the sequence of the DNA or RNA, respectively (8, 9). The advent of more affordable and available sequencing and bioinformatics platforms has enabled the determination and publication of microbial sequences with a fast turn-around time. For example, the first sequence data for SARS-CoV-2 (Risk level 3) came weeks after the first cases of a novel infectious disease were identified and the virus was isolated in Wuhan, China (10). This enabled the US Centers for Disease Control and Prevention and commercial SARS-CoV-2 RT-qPCR assays to be designed, tested and deployed with Emergency Use Authorization (EUA) by the US Food and Drug Administration (FDA) in record time.

First movers include companies and organizations that have developed a discriminator or first mover advantage, which is usually a process or product derived from intellectual property that places them ahead of their competitors. This discriminator often confers a technological edge for a first mover and early market entry, but does not guarantee market share and sustainable growth as competitors can still catch up later (11). In marketing theory, as described by Lieberman et al., first movers are often validated by leading their respective markets in sales and market share (12). First movers are also recognized by their ability to develop and commercialize leading technologies that help create new markets (13). There are several first mover theories and depending on the business case, it can be argued that although first movers have an early advantage, second movers (also called fast followers) also have advantages (14). The COVID-19 pandemic has created a niche market for first movers, fast followers, and others reacting to the need for diagnostics to inform medical care and immunological assays necessary for the research and development of vaccines and therapeutics.

In addition to pioneering a technological edge, we considered two first mover advantages by Lieberman: possessing technological leadership, and capitalizing on a feasible opportunity (12). Breakthrough technology is an innovation into a specific market space and measured according to market share. Technological leadership is sustained through discovery of those innovations that can be patented and processes and products which result in cost advantages (12). A feasible opportunity may be created among the success of that company's (and their competitors) breakthrough technology, skill, and serendipity.

Our first company, BioFire (formerly Idaho Technology, Inc.), developed a breakthrough technology using air-thermocycling to perform real time qPCR in 40 minutes which was half the time of the conventional Peltier heat block thermocyclers (15). When combined with probe chemistries such as fluorescent dyes, rapid analysis and identification of target nucleic acids can be performed. We refer to technological leadership as the action of a company visionary, often the founder, whose contribution can be measured by the number of peer-reviewed publications and patents and the ability of the company to maintain its vision. Biofire was founded in 1990 by Mr. Kirk Ririe while he was at the University of Utah, based on a feasible opportunity that arose to develop technology for the rapid detection of biowarfare agents. Together with the US Air Force as a product champion, they optimized an existing laboratory-based qPCR platform to enable development of an instrument with a rugged form factor, freeze dried reagents, automatic data analysis, and a simplified user interface. This product became the R.A.P.I.D.® System that Idaho Technology, Inc. further developed under a major DOD contract called the Joint Biological Agent Identification and Diagnostic System (JBAIDS). JBAIDS became the first biologic identification platform with FDA 510(k) clearance and a benchmark for DOD acquisition of medical devices (16). Successful performance of this contract afforded corporate growth and Idaho Technology Inc. was renamed BioFire Defense which is now an LLC of Compagnie Merieux Alliance based in the US, and BioFire Diagnostics, a subsidiary of bioMerieux. Overall, BioFire Defense has developed and deployed four different military field-forward qPCR systems (R.A.P.I.D.®, RAZOR™, JBAIDS, and FilmArray®). In addition, FilmArray® is now a Clinical Laboratory Improvement Amendments (CLIA) waived system.

Similarly, Mammoth Biosciences launched in 2018, based on CRISPR technologies developed in the lab of CRISPR pioneer Professor Jennifer Doudna from the University of California, Berkeley. She and Professor Emmanuelle Charpentier were recently awarded the 2020 Nobel Prize in chemistry for this work. The company was founded with the vision of harnessing the natural diversity of CRISPR to enable a multitude of diagnostics and therapeutics applications across biotechnology. Further advancing molecular-based detection, Mammoth Biosciences discovers and develops novel CRISPR proteins, coupled with programmable guide RNAs (gRNA), to search through genetic material from a sample for matching DNA or RNA sequences. Their DNA Endonuclease Targeted CRISPR *Trans* Reporter (DETECTR™) platform leverages specific properties of Cas protein variants that trigger indiscriminate cleavage of any nearby single-stranded nucleic acid when target sequences are recognized (17). DETECTR™ has been configured into reagents kits offering high analytical precision for use on laboratory instrumentation^2^. The company is also leveraging the DETECTR™ platform in the development of diagnostic tests for use in decentralized settings. Their mission is to improve lives by reading and writing the code of life, with a key focus on developing next-generation diagnostics that address needs from centralized to decentralized testing environments.

## Discussion

### Real World Events as Feasible Opportunities

Capitalizing on a feasible market opportunity according to Lieberman, such as a real world event, is considered a first mover advantage. In reality, investment in R&D and long-term relationships with product champions prepared both companies to respond effectively to extraordinary real world events. In 2001, following the anthrax letter attacks, BioFire Defense answered the call to provide military and civil support end users field-forward systems for rapid biodetection. A similar scenario occurred in early 2020 when the COVID-19 outbreak was just emerging and efforts by clinicians and scientists to contain and understand the disease were underway. BioFire Defense and Mammoth Biosciences, among many others, were positioned well to swiftly respond with diagnostic assays to detect SARS-CoV-2. BioFire Defense leveraged their FilmArray® platform to support COVID-19 testing for their clients in the military and defense market. The Mammoth Biosciences team developed a prototype point-of-care device for SARS-CoV-2 analogous to a standard at-home pregnancy test where the result can be visualized on a lateral flow strip (18). As of August 2020, the FDA has issued over 170 EUAs for *in vitro* diagnostics including molecular tests by BioFire Defense on March 23, 2020 and Mammoth Biosciences for their SARS-CoV-2 DETECTR™ Reagent Kit on August 31, 2020^3^.

### Shared Similarities

During our discussion, both companies expressed similar experiences and shared values in their workforce development and corporate growth. Instilling a can-do attitude and get-it-done discipline is important for start-up and small companies with limited resources along with maintaining mission focus. Strong and supportive leadership, to direct limited resources and create the vision for a long-term unique product differentiator, and the ability to develop strategic partnerships were also cited. For example, BioFire was founded with internal and external funding and expanded consistently from 40 employees in the 1990's to over 500 in 2015 when they were acquired by bioMerieux. Today, they have over 2,000 employees and the company is part of a large international company. Mammoth Biosciences founded in 2018 has raised $45M in series B venture funding, grown to over 70 employees and recently moved from an incubator laboratory space to larger facilities in the South San Francisco area. Mammoth Biosciences raised venture capital early in the company's trajectory while BioFire's early funding was from internal and external small business innovation research programs. Both companies agreed that defining priorities and focusing on project execution continues to be important and recognizing that early limited resources will be less effective when divided among competing priorities. Mammoth Biosciences also pointed out the importance of developing strong partnerships where the partners can leverage each other's strengths and technical toolboxes.

### Thriving as First Movers

Continuing research activities to discover revolutionary technologies and seeking those that are true differentiators was an attribute for both companies. Lieberman and others describe one advantage of first mover technological leadership as the ability to develop intellectual property using R&D expenditures. Anecdotally, when companies invest more of their resources toward internal R&D they tend to be more successful than those who do not. Identifying product champions especially those considered early market adopters is critical for gaining insights on product use and market behavior. While close collaborations with product champions help strengthen scientific validity and reinforce market credibility, the product champion is not necessarily a customer. It is important to distinguish between a product champion and a primary customer early on to define the expectations of each and avoid conflicts. Another observed characteristic is that business leaders tend to be very mission focused especially those at first mover companies who tend to self-invest in R&D first and acquire external funding later.

### Observations From the Panel Discussion

During the discussion “Harnessing R&D, Industry, and Entrepreneurship,” our panel aimed to link first mover advantages with our two prototypical companies. From our discussion that included one audience question that was timely and based on the need for a novel SARS-CoV-2 detection assay, we note the importance of reinforcing awareness and the need for continual funding in the area of biodefense technologies, including those that contribute to outbreak preparedness. Similar comparisons can be made from medical countermeasures, therapeutics, and vaccines and are beyond the scope of our discussion. When our panel developed this discussion, we recognized this limitation and only focused on two biotechnology companies harnessing revolutionary detection technology.

We categorized these current challenges according to first mover advantages below:

1) Current challenges exist to develop assays for emerging agents quickly, especially for those targets that are in less market demand. Some of these challenges are technical and may offer opportunities for first movers.

- Due to the *a priori* approach for designing molecular assays, there is a constant need to revise assays and test them against newly discovered strains. This so-called “signature erosion” is more apparent in agents that are rare and have significant genomic variation between isolates. The assays must be continually adapted to maintain their sensitivity and specificity when new strains arise. DETECTR™ may address this because guide RNAs are rapid to design and manufacture.
- Working on an assay for a new or rare infectious disease is complicated by the fact that it is difficult to get enough sample numbers for verification and validation testing. Contrived samples must be used in this case. With the FDA EUA, companies have more options to validate their assays in a timely and affordable manner. In addition, insurance reimbursements in clinical diagnostic market further complicates decisions on which assays to develop.
- The diagnostics market is looking for rapid molecular tests. For example, point-of-care tests to obtain a result while the patient is still in the office. For COVID-19, high-throughput molecular and serological tests are needed to determine who may be infected and to provide serological surveillance of recovered patients. There is also a demand for point-of-care tests and screening assays that can differentiate SARS-CoV-2 from other viral respiratory diseases such as influenza, other coronaviruses, and respiratory syncytial viruses.
- Perhaps someday technical breakthroughs will enable assays to be developed more quickly and address smaller markets that have been less emphasized such as those neglected tropical diseases often mentioned in global health.

2) The COVID-19 pandemic highlights the continual need to prioritize diagnostic assay development and strategic funding to invest in preparedness. Recent US Government priorities such as Health and Human Services “Operation Warp Speed” and the National Institutes of Health “RADx” create further feasible opportunities.

- Noting that private and commercial sector funding is important, US Government funding agencies such as Biomedical Advanced Research Authority (BARDA), DARPA, and USAID have incorporated cost sharing to offset investment; associated funding needs to include incentives that create and encourage private-public partnerships.
- In some respects, the clinical diagnostics market like inkjet printers is a razor and blades business model. Instruments and assays are required and complement each other where assays like razor blades and inkjet cartridges are highly consumed. Biofire Defense noted that their development and production of these multiplex assays was strengthened by an effective logistics and supply chain, highlighting their successes in markets driven by major real world events such as the outbreak of Ebolavirus in 2014 and the ongoing COVID-19 pandemic in 2020.
- US Government defense spending is valuable because it does not dilute the value of the company. Merging two funding streams is useful is the projects are overlapping e.g., COVID-19.
- Stock (shareholders) and Venture capital investment can impinge on how an assay is conceived. This is also true for government funding. Government vs. private funding also effects company evolution.
- Future technology being investigated by BioFire Defense includes “Extreme PCR,” which is a phrase coined by its inventor, Dr. Carl Wittwer (one of Idaho Technology's and BioFire's founders), and refers to techniques that can shorten qPCR runs from 10 minutes to 10 seconds (19).
- Mammoth is discovering and developing novel Cas proteins, expanding the CRISPR toolbox, and forging new partnerships in multiple applications areas across healthcare and non-healthcare verticals.

## Conclusions

The biopharmaceutical industry has a “valley of death” due to the cost, technical, and regulatory challenges associated with clinical trials. The challenges are similar in the clinical diagnostics industry and risk aversion favors the prioritization of products with higher profit margins. Outbreaks of novel infectious diseases, although relatively rare, will continue to set the stage for new first movers in diagnostics, and funding strategies with incentives to encourage industry to engage is critical. In addition, consumer demand for easy to use, point-of-care clinical diagnostics with low cost and environmental burden will continue to increase. Additionally, novel approaches to speed up testing and to reduce the environmental burden of testing, such as reusable and recyclable materials, are predicted to become an area of innovation within diagnostics.

Life science enterprises such as biopharma and biotechnology, including diagnostics, are part of the US bioeconomy that is estimated to be 2% of the US Gross National Product (20). Initiatives that support the bioeconomy include the goal of nurturing, supporting, and sustaining a domestic biosciences industry (21). During the COVID-19 pandemic, the need for diagnostic assays for molecular and serological surveillance and the demand for scalable diagnostic capabilities from both central laboratories and point-of-need locations continues to increase. First mover companies such as BioFire Defense and Mammoth Biosciences were well-positioned to tailor their molecular diagnostic assays and started developing within weeks of identification of the SARS-CoV-2 viral sequences. Biosurveillance and rapid reliable diagnostics in the midst of a pandemic ultimately saves lives. The economic consequences resulting from the COVID-19 pandemic are a reminder that the bioeconomy, particularly biotechnology and synthetic biology, remains an important component and driver of the global economy. Continual investment in R&D and successful business practices will promote resilience in this sector.

## Data Availability Statement

The raw data supporting the conclusions of this article will be made available by the authors, without undue reservation.

## Author Contributions

KY developed the panel discussion with MS, JM, and GO. KY moderated the panel discussion with MS. JM provided notes from the panel discussion. KY drafted the manuscript with contributions from MS, JM, and GO. All authors reviewed and agreed with the manuscript.

## Conflict of Interest

The authors declare that the research was conducted in the absence of any commercial or financial relationships that could be construed as a potential conflict of interest.

## Footnotes

^1^https://www.floridadaily.com/marco-rubio-bioeconomy-research-and-development-act-will-help-with-vaccines/.

^2^https://www.nibib.nih.gov/covid-19/radx-tech-program/radx-tech-phase2-awards.

^3^https://www.fda.gov/medical-devices/coronavirus-disease-2019-covid-19-emergency-use-authorizations-medical-devices/vitro-diagnostics-euas.
